# Pseudo-malignant paranasal invasive aspergillosis showing bone destruction and FDG uptake on PET/CT: A case report

**DOI:** 10.1097/MD.0000000000031759

**Published:** 2022-11-11

**Authors:** Takeshi Tsuda, Kazuya Takeda, Soichiro Fujii, Masaki Hayama, Hidenori Inohara

**Affiliations:** a Department of Otorhinolaryngology–Head and Neck Surgery, Osaka University Graduate School of Medicine, Suita City, Osaka, Japan; b Department of Otorhinolaryngology, Hyogo Prefectural Nishinomiya Hospital, Nishinomiya City, Hyogo, Japan.

**Keywords:** case report, invasive aspergillosis, paranasal mycetoma, PET/CT, pseudo-malignant paranasal invasive aspergillosis

## Abstract

Aspergillosis is a rare disease that often occurs in patients with diabetes mellitus or compromised hosts. This study describes a 60-years-old woman with unusual pseudo-malignant paranasal invasive aspergillosis with ^18^F-fluorodeoxyglucose (^18^F-FDG) uptake treated by endoscopic sinus surgery. To the best of our knowledge, there are few reported cases of paranasal fungal infection with 18F-FDG uptake.

**Patients concerns:**

A 60-years-old woman was presented with headache and nasal obstruction.

**Diagnoses:**

Computed tomography (CT) showed a shadow with bone destruction in the sinus cavity and accumulation of ^18^F-FDG uptake. The patient was diagnosed with a malignant tumor or pseudo-malignant paranasal invasive aspergillosis.

**Interventions:**

The patient underwent endoscopic sinus surgery; no neoplastic lesions were detected in the areas with CT shadows. All the observed fungal mass reservoirs were removed.

**Outcomes:**

The patient remained hospitalized for 1 week after the surgery with no significant postoperative abnormalities. There was no recurrence of the disease for 6 months, and the patient’s symptoms resolved, indicating a good course of follow-up.

**Lessons:**

Invasive aspergillosis should be considered a differential disease when positron emission tomography (PET)/CT scans show FDG uptake, CT shows bone destruction, and T2-weighted MRI scans show a low signal.

## 1. Introduction

Positron emission tomography/computed tomography (PET/CT) examinations visualize glucose metabolism in the body using radioisotope ^18^F labeled fluorodeoxyglucose (FDG). ^18^F-FDG is very effective in identifying the primary site of cancer and diagnosing invasion, metastasis, and recurrence; however, it is often inadequate for distinguishing benign from malignant lesions because it also accumulates at sites where glucose metabolism is elevated, such as inflammatory lesions.

Sinonasal mycosis is a fungal infection of the paranasal sinuses that occurs in patients with malignant tumors, and diabetes mellitus or compromised hosts such as those using systematic corticosteroids. Sinonasal mycosis can be classified into mycetoma and invasive aspergillosis in which invasive aspergillosis develops with bone destruction in the surrounding area, resulting in intracranial and intraorbital complications with poor prognosis. Due to the characteristics of this disease, it is necessary for distinguishing invasive aspergillosis from malignancy. In imaging studies, there are only few reports of PET/CT studies for sinonasal invasive aspergillosis. Herein, we report a rare case of pseudo-malignant paranasal invasive aspergillosis with ^18^F-FDG uptake.

## 2. Case report

**Figure 1.**
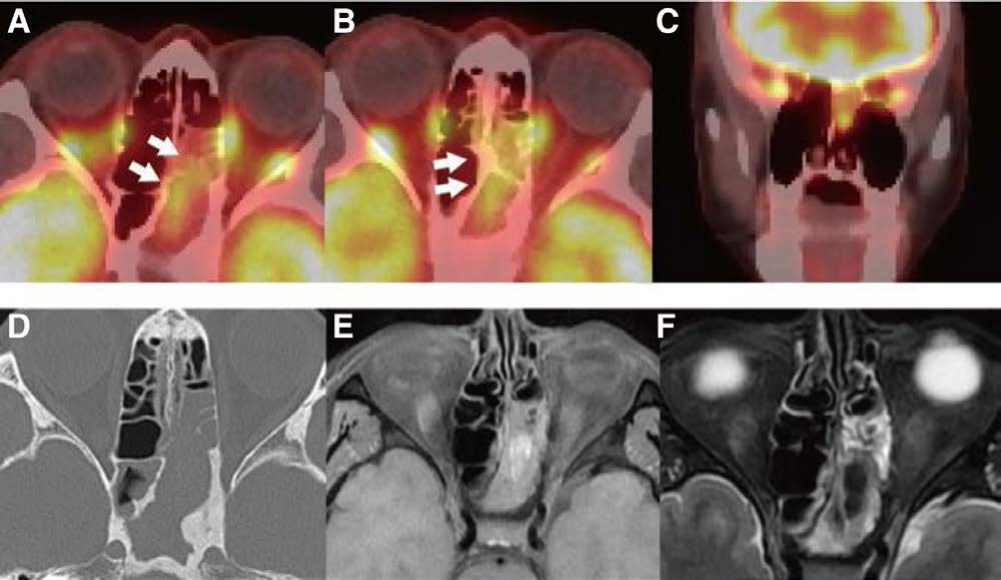
Preoperative imaging. (A–C) FDG uptake (SUVmax, 7.6) is detected in the left posterior ethmoidal and sphenoid sinuses (white arrow). (D) Axial CT scan shows bone destruction in the anterior wall of the sphenoid sinus and posterior nasal septum. In contrast, bone thickening is observed lateral to the posterior wall of the sphenoid sinus. (E and F) Mixed high-intensity and iso-intensity signal areas are observed in the lesion on T1-weighted imaging, and mixed iso-intensity and low-intensity signal areas are observed in the lesion on T2-weighted MRI scans. CT = Computed tomography, FDG = 18F-fluorodeoxyglucose, MRI = magnetic resonance imaging, SUVmax = maximum standardized uptake value.

A 60-years-old woman was presented with headache and nasal obstruction. A paranasal mass was detected in the left nasal cavity; PET/CT FDG uptake was observed in the posterior ethmoid sinus and sphenoid sinus (Fig. 1A–C). CT findings showed bone thickening and destruction in the sphenoid sinus (Fig. 1D). Magnetic resonance imaging (MRI) scans showed mixed high-intensity and iso-intensity signals in *T*1-weighed imaging and mixed iso-intensity and low-intensity signals in *T*2-weighed imaging (Fig. 1E and F). Resultantly, we performed endoscopic sinus surgery to investigate malignancy or invasive aspergillosis.

**Figure 2.**
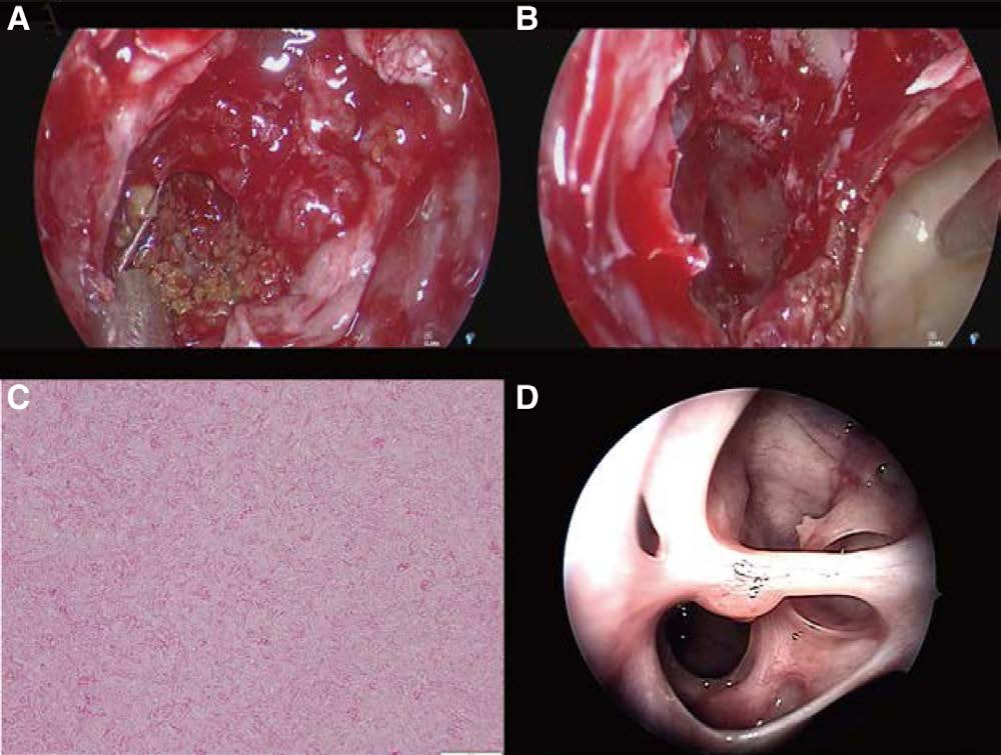
Transnasal endoscopic view of the posterior ethmoid sinus. (A) The accumulation of a suspected fungal mass from the posterior ethmoid sinus to the sphenoid sinus. (B) The region from the posterior ethmoid sinus to the sphenoid sinus after removal of the mass. (C) Histopathological analysis (hematoxylin and eosin staining) shows Y-shaped filamentous fungi with inflammatory cell infiltration. (D) Intranasal imaging at 6 months postoperatively shows no apparent recurrence.

During surgery, first the left anterior ethmoid sinus was opened followed by the basal lamella, upon which nasal endoscopy revealed a suspected fungal mass in the posterior ethmoid sinus. All the mass was removed, and there was no apparent spinal fluid leakage or orbital fat exposure (Fig. 2A and B). After the pathological examination, the diagnosis of sinus fungal Aspergillus infection was established (Fig. 2C). Her postoperative course was good, and she was discharged from the hospital on the 7th postoperative day. Six months after the surgery, her symptoms resolved and there was no evidence of recurrence (Fig. 2D).

## 3. Discussion

FDG uptake has been reported in aspergilloma of the lungs.^[1–5]^ However, few reports of FDG uptake in paranasal sinus aspergilloma have been published to date. When FDG uptake is observed, a biopsy is often necessary to confirm the presence of a malignancy or neoplasm. In the imaging diagnosis of fungal infections, CT scans frequently show calcifications that represent Aspergillus infection, and MRI shows *T*1 iso- to low and *T*2 low-to-no signal. A previous report analyzed *1*7 cases of invasive aspergillosis for which MRI information was available and reported that the frequencies of *T*1-weighted iso- or hypointense and *T*2-weighted hypointense signals were both 94%.^[6]^ Diseases with low-intensity signals on MRI *T*2-weighted scans include tumor lesions such as hematoma and melanoma.^[7,8]^ In this case, specific calcifications were not detected, however, bone destruction of the anterior wall of the sphenoid sinus and the posterior nasal septum was observed. In addition, mixed high-intensity and iso-intensity signal areas were observed in the lesion on *T*1-weighted imaging, and mixed iso-intensity and low-intensity signal areas were observed in the lesion on *T*2-weighted MRI scans. Therefore, both invasive aspergillosis and malignancy were raised as differential diseases.

In conclusion, we encountered a patient with pseudo-malignant paranasal invasive aspergillosis with ^18^F-FDG uptake. Therefore, we recommend that invasive aspergillosis should be considered a differential disease when PET/CT scans show FDG uptake, CT shows bone destruction, and *T*2-weighted MRI scans show a low signal.

## Author contributions

**Conceptualization:** Takeshi Tsuda.

**Data curation:** Takeshi Tsuda, Soichiro Fujii, Masaki Hayama.

**Supervision:** Kazuya Takeda, Hidenori Inohara.

**Writing – original draft:** Takeshi Tsuda.

**Writing – review & editing:** Kazuya Takeda.
